# The Pathological Society

**Published:** 1897-10-30

**Authors:** 


### THE PATHOLOGICAL SOCIETY.

### Simple Basic Meningitis in Infants.

The manner in which the number of the infective diseases is likely to be increased by the careful bacteriological investigation of apparently simple conditions was illustrated at the last meeting of the Pathological Society, when a paper was read by Dr. G. F. Still on the "Bacteriology of the Simple Posterior Basic Meningitis in Infants," which was shown to be a specific inflammatory lesion due to a specific microbe prevalent in England and America, and commonest during the spring. The cases observed by the author were sporadic, and were all more or less alike, the chief symptom being convulsion, retraction of the head, rigidity, opisthotonos, and hydrocephalus. The anatomical lesion found after death was thickening of the arachnoid about the base of the brain and spinal cord, with in recent cases the effusion of soft lymph. The microbe was a dipplococcus closely resembling the gonococcus.

